# Physical activity specifically evokes release of cell-free DNA from granulocytes thereby affecting liquid biopsy

**DOI:** 10.1186/s13148-022-01245-3

**Published:** 2022-02-22

**Authors:** Elmo W. I. Neuberger, Stephanie Sontag, Alexandra Brahmer, Keito F. A. Philippi, Markus P. Radsak, Wolfgang Wagner, Perikles Simon

**Affiliations:** 1 Department of Sports Medicine, Rehabilitation and Disease Prevention, Faculty of Social Science, Media and Sport, Johannes Gutenberg-University Mainz, Albert-Schweitzerstr. 22, 55128 Mainz, Germany; 2 Helmholtz-Institute for Biomedical Engineering, Stem Cell Biology and Cellular Engineering, RWTH Aachen University Medical School, Aachen, Germany; 3 Institute for Biomedical Engineering – Cell Biology, University Hospital of RWTH Aachen, Aachen, Germany; 4 Department of Medicine III, Johannes Gutenberg University Medical Center, Mainz, Germany

**Keywords:** Cell-free DNA, cfDNA, Methylation, Tissue of origin, cfDNA release, Exercise, Physical activity, Hematological malignancies

## Abstract

Physical activity impacts immune homeostasis and leads to rapid and marked increase in cell-free DNA (cfDNA). However, the origin of cfDNA during exercise remains elusive and it is unknown if physical activity could improve or interfere with methylation based liquid biopsy. We analyzed the methylation levels of four validated CpGs representing cfDNA from granulocytes, lymphocytes, monocytes, and non-hematopoietic cells, in healthy individuals in response to exercise, and in patients with hematological malignancies under resting conditions. The analysis revealed that physical activity almost exclusively triggered DNA release from granulocytes, highlighting the relevance as a pre-analytical variable which could compromise diagnostic accuracy.

**Graphical Abstract:**

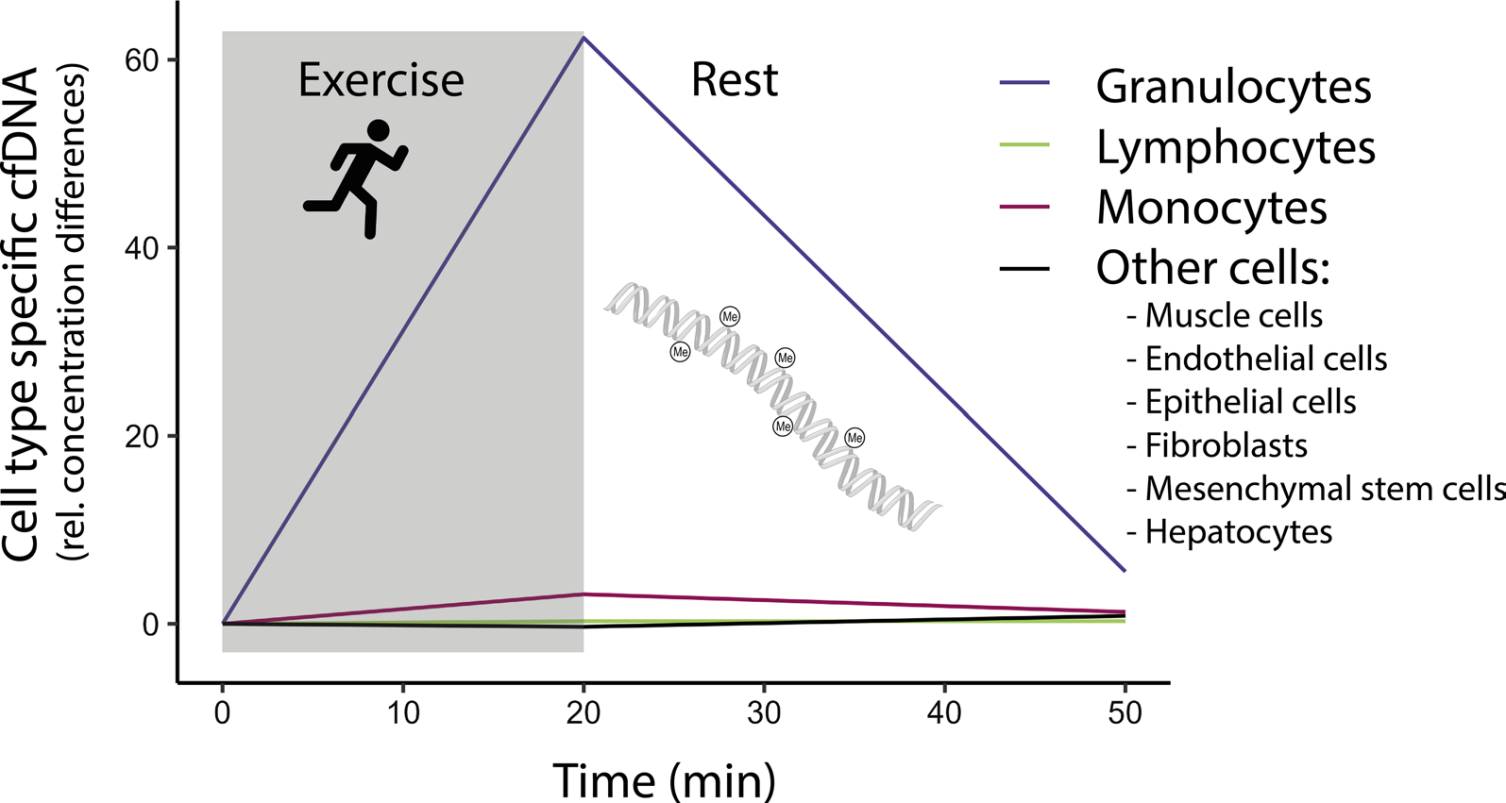

**Supplementary Information:**

The online version contains supplementary material available at 10.1186/s13148-022-01245-3.

## Introduction

Circulating cell-free DNA (cfDNA) opened up a new horizon for genomic analyses, including absolute quantification, fragmentation profile analysis, detection of mutations and copy number variations, as well as epigenetic profiling [1]. Cancers, including hematological malignancies, are accompanied by increased levels of cfDNA [2]. More recently, research indicates that DNA methylation analyses are promising tools for diagnostic and prognostic purposes, and cfDNA can be utilized for studying global methylation profiles [3, 4]. Exercise impacts immune homeostasis and leads to rapid and markedly increased levels of cfDNA in blood. Depending on exercise modality, duration, and intensity cfDNA increases 2–20 fold [5–8]. Even submaximal exercise levels lead to 2–4 fold increases [5], showing a half-life of ~ 15 min [6]. However, the origin of cfDNA during exercise has not been studied in detail and the contributing source cells remain elusive. Hence, it remains unknown how acute physical activity affects the composition of cfDNA. Exercise-induced cfDNA releases could interfere with, or improve the diagnostic accuracy of methylation specific testing, depending on whether the DNA is derived from the clinically relevant cell type.

In a sex-mismatch transplantation model we already showed that the major part of cfDNA is released from cells of the hematopoietic lineage during exercise [9]. Since the occurrence of neutrophil extracellular traps (NETs) has been described following physical exhaustion [10], and cfDNA levels are correlated with markers of neutrophil activation including neutrophil elastase, and/or myeloperoxidase [10, 11], it can be considered that granulocytes contribute to the pool of cfDNA [12]. However, a more comprehensive picture is pending. Furthermore, we have shown that a targeted CpG methylation sequencing approach can be utilized to determine the cell types of origin reliably [13, 14].

The aim of our exploratory study was to identify the source cells and kinetics of cfDNA in healthy subjects during physical activity. To this end, we used targeted methylation analysis with the pre-validated CpG sites to assess the origin of cfDNA in healthy participants with a longitudinal design—before, after, and 30 min after exercise—to consider the inter-individual variation. For comparison we also analyzed a small sub-cohort of patients with hematological malignancies under resting conditions.

## Methods

### Study collective

The study comprised of 10 healthy participants with a mean age of 26.0 ± 5.6 years and 6 patients aged 57.4 ± 11.6 with hematologic malignancies, including low risk myelodysplastic syndrome (LR MDS), chronic myelomonocytic leukemia (CMML), myelodysplastic/myeloproliferative neoplasm (MDS/MPN), myelofibrosis, and two patients with polycythemia vera (PV). All diagnoses were confirmed by a hematopathologist according to the 2016 WHO criteria [15]. The participants gave their informed consent to participate. All experimental procedures were approved by the Human Ethics Committee Rhineland-Palatinate and conformed to the standards of the Declaration of Helsinki of the World Medical Association.

### Exercise testing of healthy participants

The 10 healthy participants conducted an incremental running test on a treadmill, starting at 4 km/h. The speed was increased every three min by 1.5 km/h until volitional exhaustion as described by Ochmann et al. [16]. Venous blood samples were collected before (Pre), immediately after (Post), and 30 min after the test (+ 30′). Heart rate (electrocardiogram), as well as oxygen uptake and carbon dioxide release (spiroergometry), were recorded continuously (Geratherm Respiratory). Gas exchange and heart rate were measured continuously. After each step of the test the participants were asked for their rating of perceived exhaustion (RPE) [16].

### Sample preparation, DNA extraction and bisulfite conversion

Whole blood samples were collected in tripotassium-EDTA covered Monovettes® (Sarstedt) and were processed within < 3 h after sampling. Whole blood was centrifuged at 2500×*g* for 15 min. Separated plasma was centrifuged a second round at 2500×*g* for 15 min and stored at − 80 °C before further processing. DNA was extracted from 4 ml of plasma using QIAamp Circulating Nucleic Acid Kit (Qiagen) and eluted in 55 µl of UltraPure™ DNase/RNase-Free Distilled Water (Invitrogen). 50 µl of the eluate was bisulfite converted using the EZ DNA Methylation Kit (Zymo Research).

### Selection of cell type specific CpGs and deconvolution

Deconvolution of cell types is based on CG dinucleotides (CpGs) that are specifically hypomethylated in different cell types. Pre-validated CpG methylation sites were selected to estimate the origin of cfDNA from lymphocytes (cg17587997, FYN protooncogene (*FYN*)) [14], monocytes (cg10480329, centromere protein A (*CENPA*)) [14], and granulocytes (cg05398700, WD repeat domain 20 (*WDR20*)) [14], and to differentiate leukocytes from other cells (cg10673833, myosin IG (*MYO1G*)) [13], including endothelial cells, epithelial cells, fibroblasts, mesenchymal stem cells, hepatocytes, and muscle cells, described in Schmidt et al. [13]. We then generated a reference-based non-negative least-squares (NNLS) algorithm for the four CpGs of these cellular categories [13, 14]. An Excel calculation tool for cell type deconvolution based on the pyrosequencing measurements is provided in Additional file 1.

### Quantification of cfDNA and pyrosequencing

The cfDNA concentration was measured applying a pre-validated qPCR assay described in Neuberger et al. [17]. For pyrosequencing 4 µl of the bisulfite converted DNA were amplified with region-specific biotinylated/unmodified primer pairs (see Additional file 2) using the PyroMark PCR kit (Qiagen) according to the manufacturer’s instructions using the following protocol: Initial activation at 95 °C for 15 min, 45 cycles of 30 s at 94 °C, 30 s at 56 °C, and 30 s at 72 °C followed by a final extension at 72 °C for 10 min. Pyrosequencing was performed on the PyroMark Q96 ID with the respective reagents (Qiagen).

### Data analysis and statistics

Statistical analyses were conducted with R version 4.0.2, using tidyverse version 1.3.0, and rstatix version 0.6.0. Graphical illustrations were prepared with ggplot2 version 3.3.2 and corrplot package version 0.85. Continuous data were log 10 transformed and tested for normal distribution with Shapiro–Wilk test. Non-normal distributed data were expressed as median (25th; 75th percentiles). Global significant differences were tested with Friedman rank sum test. Paired or unpaired Wilcoxon rank sum tests with Bonferroni corrections, were used to compare within and between group differences.

## Results and discussion

Analysis of cfDNA methylation is a promising approach with potential clinical applications in the field of oncology and hemato-oncology [1]. Since physical activity increases the levels of cfDNA at low intensities [6], exercise is a relevant pre-analytical variable for liquid biopsy. It could be applied to elevate the low levels of cfDNA, but exercise could also interfere with the clinical accuracy of methylation diagnostic. Similar to other studies, we identified significantly increased absolute cfDNA concentrations in patients with myeloid neoplasms or acute leukemia [18, 19].

**Fig. 1:**
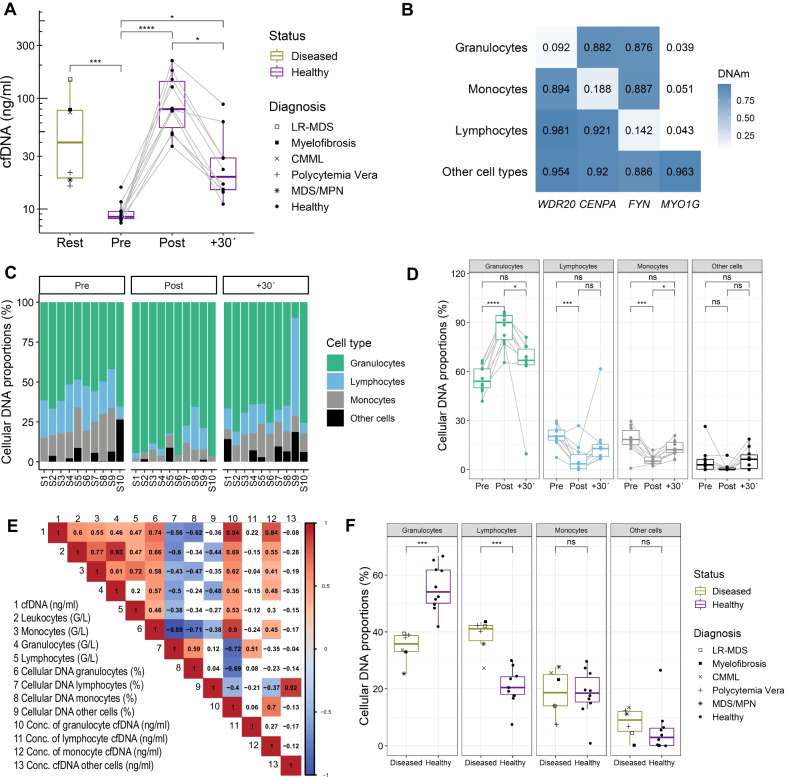
Concentration and origin of cfDNA in healthy persons during exercise and patients with hematological malignancies. **A** cfDNA concentration in blood plasma. **B** Heatmap of the percentage of methylation of the given CpGs in the reference matrix. **C**, **D** Deconvolution results indicating the origin of cfDNA samples before and after exercise in healthy subjects. **E** Spearman correlation matrix between cfDNA, blood counts, and deconvolution results. **F** Differences in cell type specific cfDNA proportions between healthy and diseased persons at rest. In all analysis *P* < .05 was considered significant. *P < .05, **P < .01, ***P < .001, ****P < .0001, ns: not significant

Patients with hematological malignancies showed elevated cfDNA concentrations of 48.1 (19.1; 78.0) ng/ml compared to 8.5 (8.1; 9.5) ng/ml in healthy untrained individuals. PV and MDS/MPN showed lower cfDNA levels than patients with LR MDS, or myelofibrosis (Fig. 1A).

During the all-out exercise test, which lasted 20.1 ± 5.4 min (mean ± SD), cfDNA levels increased ~ tenfold from 8.5 (8.2; 9.5) ng/ml to 80 (55.6; 142.7) ng/ml, decreasing to 19.8 (14.9; 28.9) ng/ml after 30 min rest, with a typical half-life of ~ 15 min [6] (Fig. 1A). Typically, healthy individuals show higher cfDNA elevations in response to exercise compared to patients, which is likely associated with exercise duration and intensity [9, 17]. However, Tug et al. studied cfDNA kinetics in hematopoietic stem cell transplantation patients showing that short-term treadmill exercise increased cfDNA levels up to sixfold, with a mean of ~ threefold [9]. 90 min after exercise the cfDNA values were reduced to resting levels again.

To estimate the cellular composition of cfDNA during exercise, we used a non-negative least squares (NNLS) deconvolution algorithm, referring to the mean DNA methylation levels of the selected CpGs from different cell types (Fig. 1B) [13]. As shown by Schmidt et al., the approach allows reliable estimation for the cellular composition by targeted analysis of the individual CpGs [13], showing similar results to previously described deconvolution results from another working group [3].

Our exploratory study demonstrates that exercise particularly triggered release of cfDNA from granulocytes. We show that granulocytes are the major source of cfDNA during exercise. Cell type specific proportions increase significantly from 54.1% (50.1; 61.8) to 90.2% (79.7; 94.4) after exercise (Fig. 1C, D). The correlation between the cell counts and cfDNA concentrations does not reflect this result (Fig. 1E). Monocyte and lymphocyte specific DNA proportions decreased significantly, but the relative DNA concentration, which was calculated by multiplying the cell type proportion with the cfDNA concentration, as measured by qPCR, indicated that a minor but significant proportion seems to be released from monocytes (see Table 1). During acute exercise and 30 min after exhaustion, no DNA was released from other cell types including muscle cells, endothelial cells, epithelial cells, fibroblasts, mesenchymal stem cells, and hepatocytes. In line with previous findings in non-exercising cohorts, granulocyte specific DNA represents the largest fraction in cfDNA, and the proportions of monocyte and lymphocyte specific cfDNA are similar, whereas the proportion from non-hematopoietic cells is lower [3]. While we provide evidence that granulocytes are the major source of cfDNA during exercise, the restricted number of represented cell types is a limitation and the inclusion of further cell types and cellular sub-characterization could be considered. The activation of granulocytes in response to exercise, as well as the fast release mechanisms of cfDNA are not clarified in detail, however, the association with oxidative stress, metabolic demands, neurohormonal stressors, acute phase proteins, and platelet activation, can be suggested [10].

**Table 1:** Hematological responses following exercise

	Pre	Post	+ 30′	Chi-square	*P*
cfDNA (ng/ml)	8.5 (8.2; 9.5)	80 (55.6; 142.7)	19.8 (14.9; 28.9)	20	4.54E−05
Leukocytes (G/L)	6 (5.6; 6.1)	9.5 (8.7; 12)	7.1 (6; 7.5)	15.8	0.000371
Monocytes (G/L)	0.4 (0.4; 0.6)	0.7 (0.6; 0.8)	0.5 (0.4; 0.6)	15.2	0.0005
Granulocytes (G/L)	3.7 (3.1; 4.3)	5.4 (4.4; 8.1)	5.1 (3.5; 5.4)	16.2	0.000304
Lymphocytes (G/L)	1.7 (1.4; 1.9)	3.1 (2.4; 3.7)	1.5 (1.3; 1.5)	18.2	0.000112
Cellular DNA monocytes (%)	18.5 (15.3; 23.9)	5.2 (3.8; 7.6)	12.2 (10.4; 16.7)	14.6	0.000676
Cellular DNA granulocytes (%)	54.1 (50.1; 61.8)	90.2 (79.7; 94.4)	66.9 (64.2; 73.8)	18.2	0.000112
Cellular DNA lymphocytes (%)	20.4 (18; 24.2)	3.5 (0.6; 9.2)	12.9 (7.9; 15.5)	11.4	0.003346
Cellular DNA other cell types (%)	3 (0.2; 6.2)	0 (0; 1)	6.3 (0.7; 9)	2.6	0.272532
Conc. of monocyte cfDNA (ng/ml)	1.7 (1.4; 2.2)	4.9 (3.6; 6.9)	3 (2.3; 4.7)	9.8	0.007447
Conc. of granulocyte cfDNA (ng/ml)	5.3 (4.4; 5.4)	67.6 (46.7; 136.1)	10.8 (9.3; 19.8)	20	4.54E−05
Conc. of lymphocyte cfDNA (ng/ml)	1.9 (1.5; 2.3)	2.1 (0.5; 8)	2.1 (1.9; 3.7)	0.6	0.740818
Conc. cfDNA other cells (ng/ml)	0.3 (0; 0.7)	0 (0; 0.8)	1.2 (0.1; 1.6)	1.4	0.496585

Data are expressed as median (25th, 75th percentiles). The relative concentration of cfDNA from different cell types was calculated by multiplying the cfDNA concentration with the cellular DNA amount (%). Global statistical differences were calculated with nonparametric Friedman rank sum test

Under resting conditions, healthy and diseased persons show similar cfDNA proportions from monocytes and other cell types, whereas the levels of granulocyte and lymphocyte specific DNA differ significantly (Fig. 1F, Table 1). The cfDNA levels of lymphocytes specific, hypomethylated cg17587997, which occur in the 5’UTR of *FYN*, are markedly increased and may be linked to pathogenesis [20]. Recently, Sontag et al. demonstrated that epigenetic lymphocyte counts in AML patients might be affected by aberrant DNA methylation in the *FYN* proto-oncogene [21]. Since epigenetic changes crucially contribute to hematological malignancies [22], further analyses of relevant methylation sites have a great potential for monitoring disease burden and treatment response.

Our exploratory study has several limitations. (1) The number of samples is relatively low, but the longitudinal analysis of the healthy donors excludes inter-individual variation. To gain further insights for which type of hemato-oncological diseases exercise might evoke additional alterations of the cfDNA profile a much larger number of patient samples would be required. Notably, our study did not aim to validate relevant methylation-based disease classifiers. (2) The age groups were not adequately matched. Since biological processes such as age can affect the methylation profile, the validity of the results can be compromised. However, in another recent study we determined the impact of age on the methylation status of cell-type specific CpGs used in this study. The stratification into younger ≤ 30 years and older ≥ 60 did not indicate significant differences [21]. However, it is conceivable that in elderly donors the exercise-induced cell-type specific release of cfDNA is slightly different and this should be further analyzed in the future. (3) We only used one exercise setting in a controlled design. To determine to what extend exercise affects cfDNA-based liquid biopsy, different clinically relevant scenarios, e.g., cycling for 30 min at moderate intensity or walking stairs, should be tested in further studies. Even low intensity physical activity with low workload, comparable to walking stairs, can increase cfDNA levels more than twofold, thereby affecting the diagnostic accuracy of cfDNA methylation based testing if resting periods are too short [9]. (4) Genome wide DNA methylation analysis might provide additional insight into the composition of other leukocyte subsets or non-hematopoietic cells [3]. The targeted analysis at cell-type specific CpGs provides a trade-off with reduced possibility for bioinformatics deconvolution and a more standardized and cost-effective approach [21].

In summary, our study provides evidence that the additional release of cfDNA during exercise is particularly attributed to granulocytes. Consequently, exercise is a relevant pre-analytical variable, which affects the composition of cfDNA. For liquid biopsy it may be advantageous to take samples at a resting state without previous exercise.

## Supplementary Information

**Additional file 1**. NNLS deconvolution table.

**Additional file 2**. Primer table.

## Data Availability

The datasets analyses of the current study are available from the corresponding author on reasonable request.

## Abbreviations

*CENPA*: Centromere protein A
cfDNA: Cell-free DNA
CMML: Chronic myelomonocytic leukemia
EDTA: Ethylenediamine tetraacetic acid
*FYN*: FYN proto-oncogene
LR MDS: Low risk myelodysplastic syndrome
MDS/MPN: Myelodysplastic/myeloproliferative neoplasm
*MYO1G*: Myosin IG
NETs: Neutrophil extra cellular traps
NNLS: Non-negative least-squares
PV: Polycythemia vera
UTR: Untranslated region
*WDR20*: WD repeat domain 20
