# Supplementary material for: Physical activity specifically evokes release of cell-free DNA from granulocytes thereby affecting liquid biopsy
Source: Clin Epigenetics. 2022 Feb 22;14:29. doi: 10.1186/s13148-022-01245-3 (PMC8864902; doi:10.1186/s13148-022-01245-3)
Supplement: Supplementary file 2 — Additional file 2. Primer table. [file 13148_2022_1245_MOESM2_ESM.docx]

| Primer | Sequence |
| --- | --- |
| *WDR20* |  |
| Forward | 5’-ACGAAGTTTAAACGAGGGTTATGTTAGGGAGAAG -3’ |
| Reverse | 5’-Biotin-GCCAACAACATTACAAAAATACCTCTTACCA-3’ |
| Sequencing | 5‘-AGATTCGGGGAGTTTTAG-3‘ |
| *FYN* |  |
| Forward | 5‘-Biotin-TAGAGGGAGGTTGTTTGGTTATAAATAGTT-3‘ |
| Reverse | 5‘-CACTTTCCAACATATTAATTATAAAAATAAATACTTTACATA-3‘ |
| Sequencing | 5‘-CTAATTTACAAACAAATAACAC-3‘ |
| *CENPA* |  |
| Forward | 5‘-Biotin-ATTATGTTGGTTAGGTTGGTTTTTAATTGTTAATT-3‘ |
| Reverse | 5‘-AAAATCAAAAAAACACTCCTACCCTTTCTCTTA-3‘ |
| Sequencing | 5‘-AAACACTCCTACCCTTTCTC-3‘ |
| *MYO1G* |  |
| Forward | 5‘-TGTTGTTAGGGTTGGAAGTTAATTT-3‘ |
| Reverse | 5‘-Biotin-CACCAACCTCCTCCAATACTAATATAA-3‘ |
| Sequencing | 5‘-GGGGAGGATTTAGT-3‘ |
|  | |

**Supplemental Table**: Primers used for pyrosequencing.
